# *Coriolus versicolor* extracts: relevance in cancer management

**Published:** 2008-04

**Authors:** M. Szeto

**Affiliations:** Master of Science in Clinical Nutrition, University of Medicine and Dentistry of New Jersey, School of Health Related Professions, Newark, New Jersey, U.S.A

**Keywords:** Complementary and alternative medicine, natural health products, *Coriolus versicolor*, CV extracts, polysaccharide-peptide, polysaccharide-K, mushrooms

URL: http://www.current-oncology.com/index.php/oncology/article/view/147/209

## E-JOURNAL LINKED ABSTRACT

The use of complementary and alternative medicine is gaining popularity worldwide. Cancer patients are major consumers of natural health products for a variety of reasons; the most common is to build the body’s defences by augmenting the immune system. Various species of mushrooms have been studied for decades because of their alleged immunostimulating properties. Active substances from more than fifty species of mushrooms have been isolated and found to have such properties. Among those substances, polysaccharide-K and polysaccharide-peptide extracted from the mushroom *Coriolus versicolor* (CV), have been more systematically investigated in human cancer research. Extracts of CV are extremely popular in certain ethnic communities with a long tradition of employing healing practices that are unconventional as compared with Western medical approaches. Cancer patients are using CV extracts as part of their adjunctive cancer therapy. Cancer specialists and allied health care professionals may not be fully aware of such a choice, especially when patients choose not to disclose the information. As communities in North America become more culturally diverse, a rise in the use of natural health products is probably inevitable. Better understanding of some popular natural health products and the reasons behind their use may foster communication between patient and health care provider, and such communication is crucial in helping patients to address their needs and concerns with regard to the hope of optimizing cancer care. Exploring the mechanisms of CV extracts, their safety, their risks. and their possible benefits is one step toward those goals.

